# Osteoporosis diagnosis and ingredients of prescription medications: a population-based study

**DOI:** 10.3389/fphar.2025.1522937

**Published:** 2025-07-11

**Authors:** Xiaohong Huang, Zhendong Feng, Xiaohua Li, Dongxu Zhu, Yingze Zhang

**Affiliations:** ^1^ Shandong Institute of Traumatic Orthopedics, Medical Research Center, The Affiliated Hospital of Qingdao University, Qingdao, China; ^2^ National Institute on Drug Dependence and Beijing Key Laboratory of Drug Dependence, Peking University, Beijing, China; ^3^ Department of Pathology, Weifang People’s Hospital, Weifang, China; ^4^ School of Medicine, Nankai University, Tianjin, China; ^5^ Department of Orthopedics, The Affiliated Hospital of Qingdao University, Qingdao, China; ^6^ Department of Orthopedics, The Third Hospital of Hebei Medical University, Shijiazhuang, China

**Keywords:** osteoporosis diagnosis, fragility fractures, active pharmaceutical ingredients, rational drug use, disease management

## Abstract

**Background:**

Osteoporosis (OP) is common in the elderly, who typically have multiple comorbidities. Current guidelines for managing drug-induced OP are limited due to the complexity of multi-agent medications and the lack of sufficient clinical data.

**Methods:**

Information of demographics, health status, prescription medication use, OP diagnoses, and bone fracture history in US adults aged ≥50 years was from NHANES. Administration of individual medication ingredients was extracted and association between medication component use and OP diagnosis was determined. National trends in OP diagnosis, prescription medication use, and medication ingredient administrations were examined.

**Results:**

OP diagnosis prevalence rose from 9.00% to 13.23% during 1999–March 2020 (p-trend = 0.00). Increased medication prescription was noted in OP patients (p-trend_No. prescription medications=4–7_ <0.0001, p-trend_No. prescription medications≥8_ < 0.0001, and p-trend_Days taking medications≥500_ < 0.0001). Thirty-four medication ingredients were correlated with OP diagnosis, including three OP-specific medications, three avoided in OP patients in current practice, seven contribute to OP but commonly prescribed, four relieved OP when treating diseases causing secondary OP, two bone health-friendly agents, and 15 lack of prior statistical records to support their clinical use in OP. Amongst 10 ingredients associated with OP diagnosis may be underlying their roles in regulating bone remodeling, sympathetic activity, and gastric acidity, whereas the remaining five were not clear.

**Conclusion:**

The findings of this study contribute to updating and improving the existing guidelines. Efforts are recommended to examine how the use of medications contribute to OP and to identify alternative treatments for comorbidities.

## Graphical Abstract

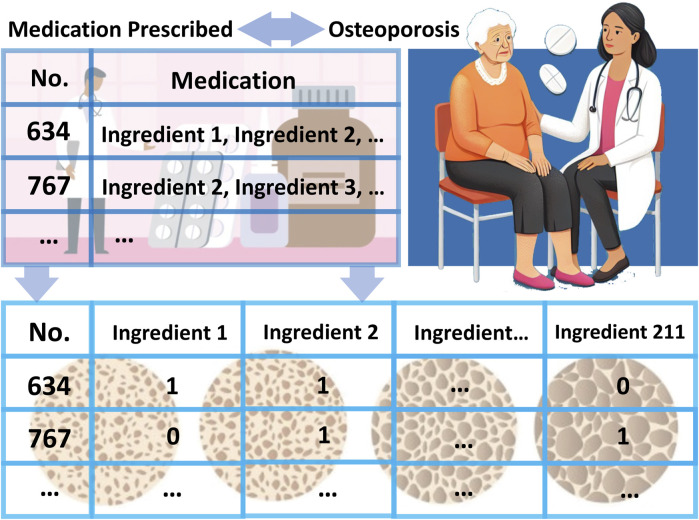

## Introduction

As the global population ages, the number of fractures is expected to increase—by 310% from 1990 to 2050 (Gullberg et al., 1997)—leading to heightened morbidity and mortality. Annual fractures and costs are expected to increase by almost 50% by 2025 (Burge et al., 2007). Osteoporosis (OP), characterized by reduced bone mass and compromised bone structure, is the leading cause of fractures in the elderly. Moreover, Centers for Disease Control and Prevention (CDC) reported that 76.9% of Medicare and Medicaid recipients aged 65 and older have multiple chronic conditions (Peter et al., 2020). And prescription medications used to treat diseases can further elevate the risk of OP via bone–organ axes (Deng et al., 2024; Foessl et al., 2023). Particularly, most medicines contain multiple active substances to increase their effectiveness, to target different aspects of the disease, and or to simultaneously relieve several symptoms, which make it complex and difficult to manage drug-induced OP. However, drug holiday or switching to bone health-friendly medications is recommended but not always feasible, attributing to the limited clinical data and unclear mechanisms.

Dealing with the modifiable factors, such as avoiding specific OP risk associated ingredients or carefully including them in combined pharmacotherapy, is crucial for effective OP prevention and treatment. The aim of this study is to determine the association between the active pharmaceutical ingredient use and OP diagnosis, and the finding of this study will contribute to develop rational drug use strategies for OP management. Specifically, this study 1) examined the national trend in OP diagnoses; 2) analyzed the variety and duration of medication prescribed in the OP diagnosed population; 3) determined the association between drug ingredient use and OP diagnosis and tracked national trend in the related ingredient use.

## Materials and methods

### Database and study population

Participants in National Health and Nutrition Examination Survey (NHANES), a nationally representative survey conducted in 2-year cycles from 1999 to March 2020, provided written informed consent, and the National Center for Health Statistics Research Ethics Review Board approved the study protocols. This study followed the Strengthening the Reporting of Observational Studies in Epidemiology (STROBE).

**FIGURE 1:**
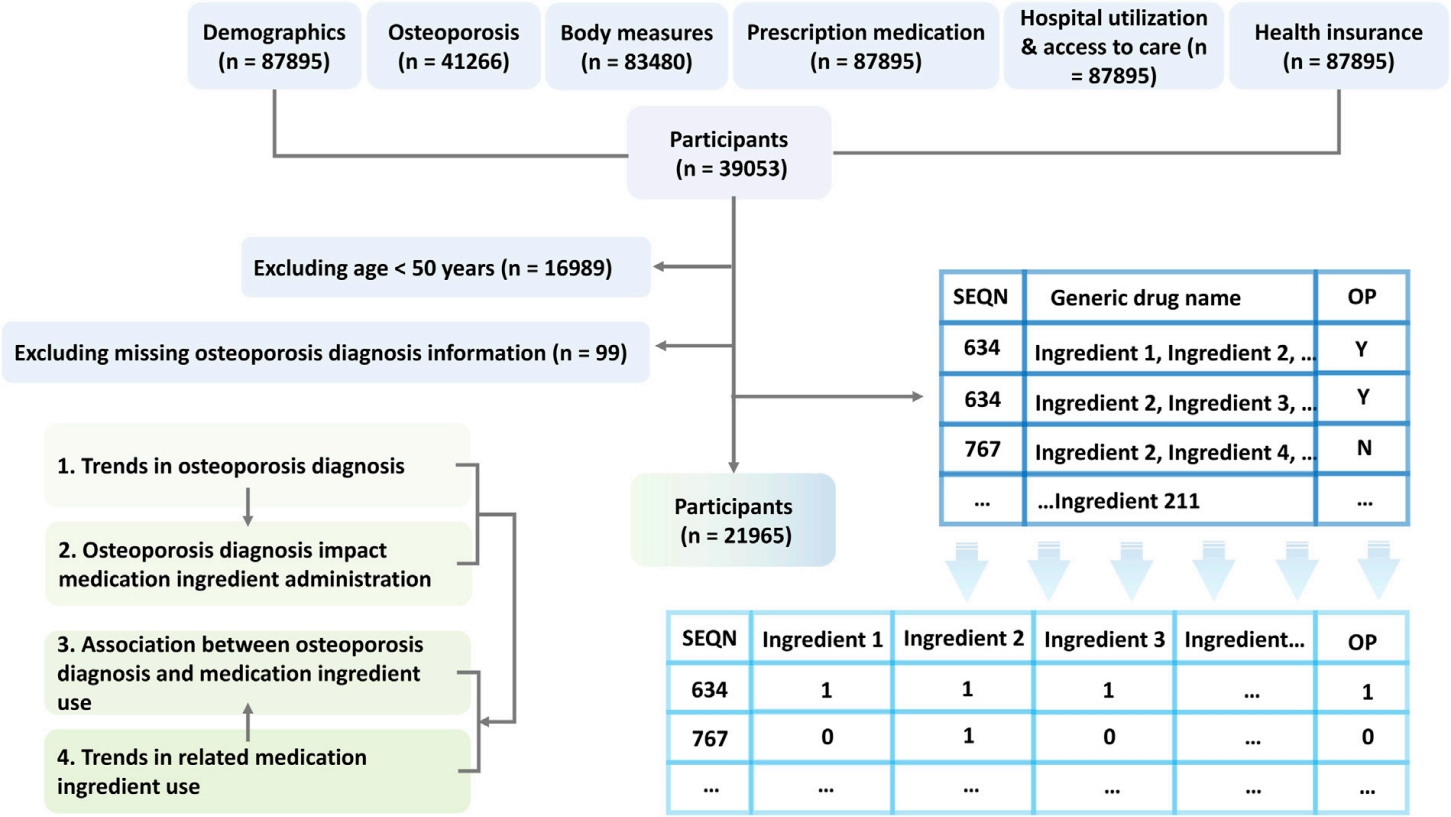
Participants, study design, and prescription medication ingredient identification.

Our analysis focused on cycles with complete records in demographics, osteoporosis, body measures, prescription medication, hospital utilization and access to care, and health insurance, as shown in Figure 1. Thus, eight cycles (1999–2000, 2001–2002, 2003–2004, 2005–2006, 2007–2008, 2009–2010, 2013–2014, 2017–March 2020) were included in this study. The study sample was limited to adults aged 50 and older with a definite answer regrading OP diagnosis (yes/no).

### Prescription medication and individual medication ingredient administration

Data on prescription medication use, including generic drug name, No. prescription medications, and days taking medications, were obtained from “prescription medication” questionnaires. The individual ingredients of these drugs were extracted from the records under “generic drug name”, as shown in Figure 1. These ingredients were then categorized into therapeutic classes based on the Multum Lexicon Plus drug database (Centers for Disease Control and Prevention CDC, 2007).

### Osteoporosis diagnosis and bone fracture history

Based on the responses to the question “Ever told had osteoporosis/brittle bones (yes/no)”, individuals diagnosed with OP were classified into the OP group, while those who explicitly stated they had not been diagnosed with OP were categorized into the non-OP group. In addition, bone fracture history was determined by aggregating reported cases of hip, wrist, and vertebral fractures.

### Clinical, demographic and socioeconomic characteristics

Clinical and demographic information, including age, sex, body mass index (BMI), race/ethnicity, and self-reported health status, were collected from standardized questionnaires and physical examinations. BMI categories were defined as follows: underweight (<18.5), normal weight (18.5–24.9), overweight (25–29.9), and obese (≥30). Because postmenopausal women with low BMI exhibit osteopenia with predisposition for OP, and fat mass assumes a determining role in predicting the bone mineral density (BMD) of the lumbar vertebrae and femoral neck of postmenopausal women (Wu and Du, 2016). Race/ethnicity analyzed included non-Hispanic white, non-Hispanic black, Hispanic, and Other (including multiple races). This assessment was conducted because of the recognized racial and ethnic disparities in the risk and incidence of OP (Thomas, 2007). Individuals with a confirmed diagnosis may report a lower self-assessment of their health, whereas those who view their health more positively may be underdiagnosed. Therefore, the current health status of the participants was analyzed, self-reported as “excellent or very good”, “good”, or “fair or poor”.

Socioeconomic information, including education level, family income to poverty ratio (PIR), and insurance status, which reflects the medication adherence and accessibility within the population, was collected using standardized questionnaires. The education level of the household head was categorized as less than a high school degree, a high school degree, or higher than a high school degree. PIR was calculated as the ratio of family income to the poverty threshold and categorized as <1, 1–1.9, 2–2.9, 3–3.9, or ≤4. Insurance status was self-reported as either insured (including public and private sources) or uninsured. These characteristics were evaluated to assess the socioeconomic status of households in relation to medication use (Venkatesh et al., 2019; McCabe et al., 2023).

### Statistical analysis

Prevalence rates and 95% confidence intervals (CIs) were reported for categorical variables. The chi-square (χ2) test was used to evaluate the consistency of distributions of categorical covariates between OP and non-OP groups, including the clinical, demographic and socioeconomic characteristics.

Trends of OP diagnosis across the survey cycles and OP prevalence by bone fracture history, No. prescription medications, and days taking prescription medications were calculated using a linear regression model. The combined survey cycle was considered as a continuous variable.

To determine medication strategy influenced by OP diagnosis, logistic regression was used to analyze the association between OP diagnosis and medication ingredient administration. The threshold for statistical significance was set at p < 0.05. Odds ratios (ORs) with 95% CIs were derived from a multivariable logistic regression model to assess the altered medication administration by OP diagnosis. National trends in the prescription of specific medication ingredients associated with OP diagnosis were then outlined using linear regression, adjusting for education level, RIP, and insurance status.

To assess the robustness of the association results, sensitivity analyses were performed by 1) assessing the change in use of each ingredient by OP diagnosis, 2) excluding adults aged ≥80 years or with a BMI ≥30 kg/m^2^ who were more prone to multiple comorbidities.

Data analysis for this study applied rigorous methods tailored to structured survey data, including stratification, clustering, and weighting to ensure nationally representative estimates. SAS software (version 9.4) was used, with statistical significance set at p < 0.05. Python (version 9.3) was used to generate diagrams.

## Results

### Characteristics of study population

This study identified a final sample size of 21,965 individuals, representing 669,561,146 noninstitutionalized US adults aged 50 and older (Supplementary Table S1). Among them, 2,408 individuals were diagnosed with OP, constituting 11.06% [95% CI, 10.38%–11.75%] of the participants (Table 1). Among OP patients, 24.11% [95% CI, 22.10%–26.11%] had a history of bone fractures, 19.43% (17.70–21.16) were aged 80–89 years, and 30.26% [95% CI, 27.95%–32.58%] were of obesity. OP patients reported similar distributions across health status categories: excellent or very good (33.00% [95% CI, 30.10%–35.90%]), good (34.68% [95% CI, 32.10%–37.26%]), and fair or poor (32.32% [95% CI, 29.46%–35.18%]). OP patients had a higher prevalence of using ≥8 prescription medications compared to non-OP individuals (36.86% [95% CI, 33.96%–39.76%] vs. 23.22% [95% CI, 22.12%–24.32%]), as well as a longer duration of taking prescription medications (64.04% [95% CI, 61.07%–67.00%] vs. 51.76% [95% CI, 50.48%–53.03%]). And the vast majority were insured, with a rate of 96.18% [95% CI, 95.02%–97.34%] in OP group (Supplementary Table S2).

**TABLE 1:** Clinical and demographic characteristics by osteoporosis diagnosis among US adults aged 50 and older, 1999–March 2020^a^.

Characteristic	% (95% CI)		p–value^b^
	OP	Non-OP	
Unweight sample, No.	2408	19557	NA
Weighted sample, No.^c^	74082254	595478892	NA
Bone fracture history			
Yes	24.11 (22.10–26.11)	13.52 (12.74–14.30)	<0.0001
No	75.89 (73.89–77.90)	86.48 (85.70–87.26)	
Age, y			
50–59	21.59 (18.99–24.19)	44.94 (43.73–46.15)	<0.0001
60–69	29.51 (27.04–31.97)	29.54 (28.50–30.58)	
70–79	29.47 (27.43–31.52)	17.18 (16.51–17.85)	
≥80	19.43 (17.70–21.16)	8.35 (7.80–8.89)	
Sex			
Male	11.61 (10.10–13.13)	50.97 (50.28–51.65)	<0.0001
Female	88.39 (86.87–89.90)	49.04 (48.35–49.72)	
Race/ethnicity			
Non-Hispanic white	82.25 (79.55–84.95)	75.57 (73.05–78.09)	<0.0001
Non-Hispanic black	5.53 (4.45–6.61)	10.26 (8.84–11.67)	
Hispanic	7.11 (5.45–8.77)	8.62 (7.16–10.08)	
Other	5.11 (3.83–6.39)	5.56 (4.78–6.33)	
BMI, kg/m^2^			
Underweight <18.5	5.51 (4.35–6.67)	3.10 (2.71–3.48)	<0.0001
Normal weight 18.5–24.9	33.92 (31.61–36.22)	23.66 (22.68–24.63)	
Overweight 25–29.9	30.31 (27.99–32.62)	35.77 (34.81–36.74)	
Obese ≥30	30.26 (27.95–32.58)	37.47 (36.20–38.75)	
Self-reported health status			
Excellent or very good	33.00 (30.10–35.90)	44.97 (43.56–46.37)	<0.0001
Good	34.68 (32.10–37.26)	33.50 (32.52–34.48)	
Fair or poor	32.32 (29.46–35.18)	21.53 (20.47–22.59)	
No. prescription medications			
1–3	24.02 (21.41–26.62)	38.99 (37.66–40.32)	<0.0001
4–7	39.12 (36.48–41.76)	37.79 (36.71–38.87)	
≥8	36.86 (33.96–39.76)	23.22 (22.12–24.32)	
Days taking medications			
0/refused/missing	8.77 (7.33–10.20)	24.80 (23.86–25.74)	<0.0001
<500	27.19 (24.36–30.03)	23.45 (22.48–24.41)	
≥500	64.04 (61.07–67.00	51.76 (50.48–53.03)	

^a^ Data from NHANES., data are present as prevalence, % (95% CI) unless indicated otherwise.

^b^ Calculated with χ2 test to determine the consistency of categorical distribution of variables between OP and non-OP groups.

^c^ Data are weighted to be nationally representative.

### Trends in osteoporosis diagnosis and prescription medication use

**FIGURE 2:**
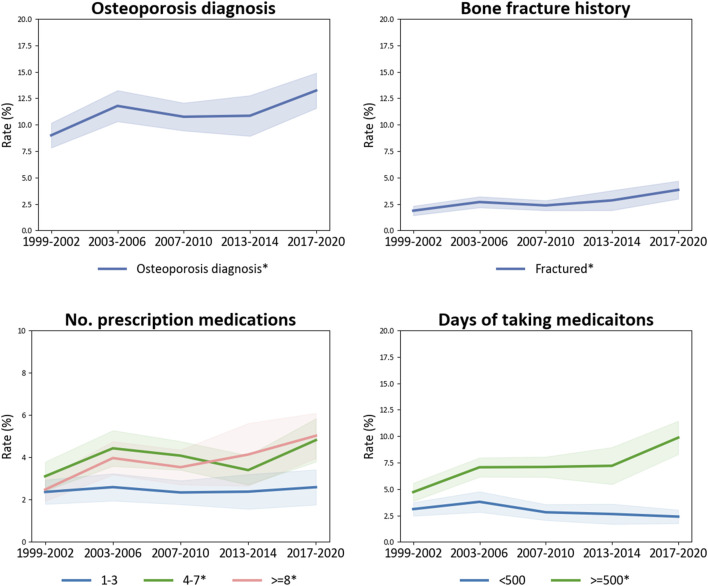
Trends in osteoporosis diagnosis among US adults aged 50 years and older, 1999–March 2020 and trends of bone fractures, No. prescription medications used, and days taking medications in the elderly diagnosed with osteoporosis. Abbreviations: BMI, body mass index. *The prevalence is significantly altered.

The prevalence of OP diagnosis increased from 9.00% [95% CI, 7.83%–10.17%] in the 1999–2002 cycle to 11.78% [95% CI, 10.31%–13.25%] in the 2003–2006 cycle, decreased to 10.75% [95% CI, 9.44%–12.06%] in the 2007–2010 cycle, plateaued until the 2013–2014 cycle and then increased again to 13.23% [95% CI, 11.57%–14.89%] in the 2017–March 2020 cycle (Figure 2; Supplementary Table S3). The prevalence of OP patients with a history of sustained bone fractures increased from 1.87% [95% CI, 1.43%–2.32%] to 3.84% [95% CI, 3.00%–4.68%].

The prevalence of prescription medication use in the OP diagnosed population was increased, indicated by an rising trend in OP patients using ≥8 medications, rising from 2.48% [95% CI, 1.94%–3.01%] in the 1999–2002 cycle to 5.02% [95% CI, 3.95%–6.90%] in the 2017–March 2020 cycle (p-trend = 0.00), and the increase in the proportion of elderly adults who taking prescription medications for more than 500 days (p-trend <0.0001), from 4.72% [95% CI, 3.86%–5.57%] to 9.86% [95% CI, 8.29%–11.43%].

### Osteoporosis diagnosis and medication ingredient administration

**FIGURE 3:**
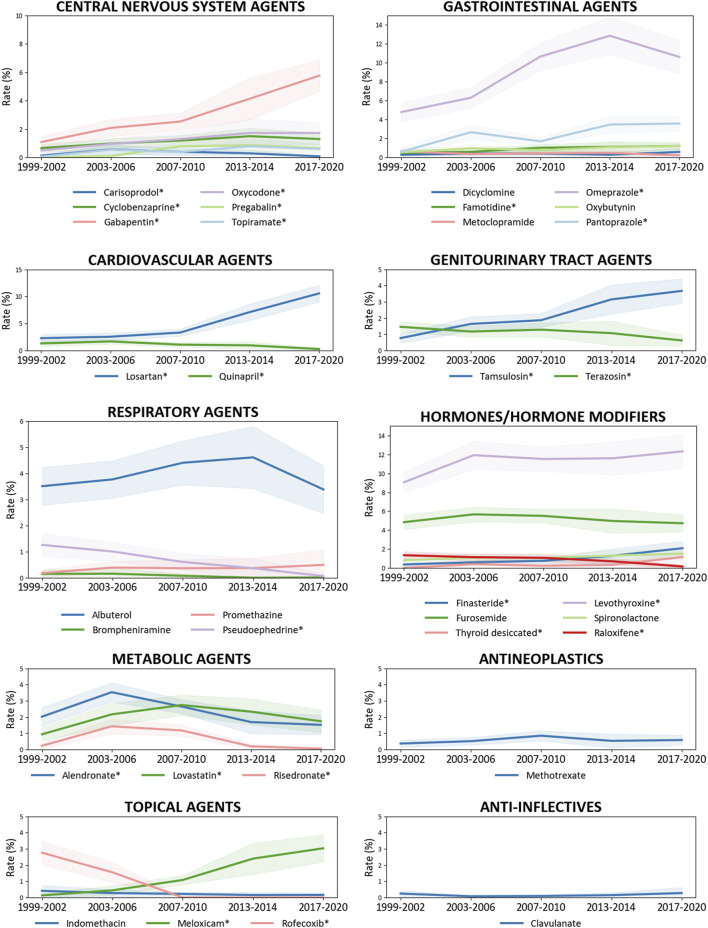
Trends in the administration of prescription medication ingredients related to osteoporosis diagnosis among US adults aged 50 years and older, 1999–March 2020. The prescription medications are categorized into therapeutic classes using the Multum Lexicon Plus drug database. National trends in the prevalence of corresponding medication ingredients in Table 2 are determined by linear regression in Supplementary Table S4. *The prevalence is significantly altered.

Significant correlations were found between OP diagnosis and 34 medication ingredients, among the 211 ingredients extracted from the “generic drug name” field, categorized into 22 subcategories across 10 agents (Table 2). And the national trends of these ingredients were explored further (Figure 3; Supplementary Table S4). Of these, 7 ingredients were used sparingly in osteoporotic patients, including an anticonvulsant topiramate (OR 0.18 [95% CI, 0.09–0.37], p < 0.0001), an angiotensin-converting enzyme inhibitor (ACEI) quinapril (OR 0.59 [95% CI, 0.38–0.92], p = 0.02), a nasal decongestant pseudoephedrine (OR 0.26 [95% CI, 0.10–0.69]), p = 0.01), two alpha-blockers tamsulosin (OR 0.37 [95% CI, 0.21–0.67], p = 0.00) and terazosin (OR 0.40 [95% CI, 0.22–0.72], p = 0.00), a 5-alpha reductase (AR) inhibitor finasteride (OR 0.51 [95% CI, 0.27–0.95]), p = 0.04), a non-steroidal anti-inflammatory drug (NSAID) indomethacin (OR 0.15 [95% CI, 0.05–0.47], p = 0.00); and, the remaining 27 ingredients were commonly used in OP cases. Among them, 13 ingredients showed increasing trends, including cyclobenzaprine (p-trend = 0.01), gabapentin (p-trend <0.0001), oxycodone (p-trend = 0.00), losartan (p-trend <0.0001), famotidine (p-trend = 0.01), pantoprazole (p-trend <0.0001), omeprazole (p-trend <0.0001), oxybutynin (p-trend = 0.05), (p-trend <0.0001), tamsulosin (p-trend <0.0001), finasteride (p-trend <0.0001), thyroid desiccated (p-trend <0.0001), levothyroxine (p-trend = 0.00), and meloxicam (p-trend = <0.0001); six showed decreasing trends, including quinapril (p-trend = <0.0001), pseudoephedrine (p-trend = <0.0001), brompheniramine (p-trend = 0.05), terazosin (p-trend = 0.00), raloxifene (p-trend = <0.0001), rofecoxib (p-trend = <0.0001); the prevalence of six ingredients initially increased and decreased in recent years, including carisoprodol (p-trend = 0.00), pregabalin (p-trend <0.0001), topiramate (p-trend <0.0001), lovastatin (p-trend <0.0001), alendronate (p-trend <0.0001), risedronate (p-trend <0.0001); and, the consumption of remind nine ingredients remained stable (p-trend >0.05). Through the 2017–March 2020 cycle, levothyroxine (12.33% [95% CI, 10.54%–14.12%]) in the hormones/hormone modifiers, omeprazole (10.62% [95% CI, 8.86%–12.38%]) in the gastrointestinal agents, and losartan (10.58% [95% CI, 9.05%–12.11%]) in the cardiovascular agents were among the most common OP-related ingredients administrated to 10% of US adults aged 50 and older.

**TABLE 2:** Association between osteoporosis diagnosis and prescription medication ingredient administration among US adults aged 50 and older, 1999–March 2020^a^ ^,^ ^b^.

Prescription medication^c^	Univariate^d^	Multivariate^e^	
	OR (95% CI)	OR (95% CI)	p-value
CNS agents			
Skeletal muscle relaxants			
Carisoprodol	3.72 (1.68–8.23)	2.66 (1.14–6.22)	0.02
Cyclobenzaprine	3.45 (2.31–5.17)	2.44 (1.56–3.83)	0.00
Anticonvulsants^f^			
Pregabalin	2.50 (1.41–4.44)	2.04 (1.07–3.91)	0.03
Topiramate	0.53 (0.24–1.15)	0.18 (0.09–0.37)	<0.0001
Gabapentin	2.76 (2.15–3.55)	1.90 (1.41–2.55)	<0.0001
Narcotic analgesics			
Oxycodone	2.58 (1.77–3.75)	2.07 (1.30–3.32)	0.00
Gastrointestinal agents			
H2-blockers			
Famotidine	2.12 (1.35–3.33)	2.23 (1.36–3.66)	0.00
Prokinetics			
Metoclopramide	2.82 (1.58–5.03)	2.15 (1.15–4.01)	0.02
PPIs^f^			
Pantoprazole	1.96 (1.47–2.60)	1.56 (1.12–2.17)	0.01
Omeprazole	1.74 (1.49–2.02)	1.28 (1.00–1.63)	0.05
Anticholinergics			
Dicyclomine	3.52 (1.80–6.91)	2.25 (1.21–4.16)	0.01
Oxybutynin	3.25 (2.01–5.24)	2.28 (1.40–3.71)	0.00
Cardiovascular agents			
ARBs			
Losartan	1.69 (1.35–2.11)	1.55 (1.21–1.99)	0.00
ACEIs			
Quinapril	0.79 (0.46–1.36)	0.59 (0.38–0.92)	0.02
Genitourinary tract agents			
Alpha blockers			
Tamsulosin	0.49 (0.29–0.80)	0.37 (0.21–0.67)	0.00
Terazosin	0.45 (0.25–0.79)	0.40 (0.22–0.72)	0.00
Respiratory agents			
Nasal decongestants			
Pseudoephedrine	0.89 (0.45–1.79)	0.26 (0.10–0.69)	0.01
Bronchodilators			
Albuterol	2.13 (1.72–2.64)	1.41 (1.05–1.90)	0.02
Antihistamines			
Promethazine	4.95 (2.09–11.74)	4.88 (1.32–18.03)	0.02
Brompheniramine	3.05 (0.72–13.01)	7.90 (1.64–38.08)	0.01
Hormones/hormone modifiers			
AR inhibitors			
Finasteride	0.37 (0.18–0.80)	0.51 (0.27–0.95)	0.04
Furosemide	1.95 (1.62–2.33)	1.39 (1.08–1.79)	0.01
Thyroid hormones^f^			
Thyroid desiccated	2.24 (1.01–4.97)	2.85 (1.06–7.67)	0.04
Levothyroxine	2.31 (2.02–2.64)	1.82 (1.54–2.15)	<0.0001
Mineralocorticoid receptor antagonists			
Spironolactone	2.25 (1.53–3.33)	1.69 (1.04–2.75)	0.04
SERMs			
Raloxifene	7.06 (4.78–10.42)	6.69 (4.30–10.40)	<0.0001
Metabolic agents			
Statins^g^			
Lovastatin	1.80 (1.29–2.51)	1.69 (1.15–2.49)	0.01
Bone resorption inhibitors			
Alendronate	28.44 (21.87–36.97)	33.73 (24.79–45.89)	<0.0001
Risedronate	18.78 (12.02–29.34)	22.02 (13.42–36.15)	<0.0001
Antineoplastics^f^			
Antimetabolites			
Methotrexate^f^	4.16 (2.49–6.95)	2.09 (1.02–4.28)	0.04
Topical agents			
NSAIDs			
Indomethacin	0.23 (0.07–0.76)	0.15 (0.05–0.47)	0.00
Meloxicam	2.60 (1.81–3.74)	1.80 (1.18–2.73)	0.01
Rofecoxib	2.66 (1.75–4.06)	2.48 (1.54–3.98)	0.00
Anti-infectives			
Beta-lactamase inhibitors			
Clavulanate	2.32 (0.74–7.29)	5.30 (1.27–22.14)	0.02

^a^ Data from NHANES.

^b^ Name of the medication ingredients is based on the NHANES records.

^c^ The medication ingredients are categorized into therapeutic classes based on the Multum Lexicon Plus drug database.

^d^ Univariable logistic regression is used to control for correlation between individual drug and OP.

^e^ Multivariable logistic regression is used to control for correlations among various risk factors. Data are present as OR (95% CI), and p-value is interpreted as a measure of statistical evidence. Medication ingredients with statistical significance obtained from multivariable logistic regression were presented; note that, uncertainty in the distribution of the outcome has been excluded despite a p-value <0.05 from multivariate regression.

^f^ Listed as a risk factor in OP guideline (LeBoff et al., 2022).

^g^ Stains are also known as HMG-CoA reductase inhibitors.

### Sensitivity analysis

The associations between OP diagnosis and administration of individual drug ingredients remained robust, as the direction and magnitude of most changes in the medication ingredient administration remained in the crude weighted logistic regression model adjusted for a single ingredient (Table 2) and in the multivariable model in a population excluding elderly aged ≥80 years (Supplementary Table S5) or obese individuals (Supplementary Table S6).

## Discussion

This study updates the national trend in OP diagnosis and highlights the rising trends in fragility fractures and medications prescribed for OP patients, underscoring the need for vigilance in preventing drug-induced OP. And the prevalent medication ingredients in this study emphasize the involvement of the bone–thyroid, bone–gastrointestinal, and bone–cardiovascular axes in OP development, hinting the importance of comorbidities management and rational drug administration in the elderly.

### Based on clinical reports

Though the OP diagnosis was increased, the use of bisphosphonates (alendronate, risedronate) and raloxifene, the FDA-approved OP drugs (LeBoff et al., 2022), was decreased. This may be due to the emergency of anabolic and catabolic treatments such as denosumab (Prolia, 2010), abaloparatide (Tymlos, 2017), and romosozumab (Evenity, 2019) (Kuril et al., 2024). Except for the OP drugs, the ingredients identified in this study can be classified as follows, based on the clinical reports bonding medical application and OP as well as the national trends in medication prescribed:

• Avoided in OP patients due to their adverse effects on bone formation or bone fracture healing, including topiramate, indomethacin, and rofecoxib (Zheng et al., 2020; Heo et al., 2011).
• Increased OP or fracture risk but are commonly used, including methotrexate, thyroid desiccated, levothyroxine, furosemide, gabapentin, pregabalin, and omeprazole (Ricciardi et al., 2013; Sakr, 2024; Jørgensen et al., 2023; Roux et al., 2009).
• Relieve OP symptoms, treat diseases that induced secondary OP, and or treat OP complications, including oxycodone used in osteoporotic pain management (Ali et al., 2024), losartan and quinapril used to treat hypertension and diabetic nephropathy (Huang and Ye, 2024; Liu et al., 2020; Barkhordarian et al., 2023), and clavulanate used in anti-infection.
• Bone health-friendly agents, including spironolactone and famotidine (Song et al., 2024; Haddadi et al., 2024).
• Lack of clinical supports, including carisoprodol, brompheniramine, promethazine, dicyclomine, oxybutynin, albuterol, pseudoephedrine, pantoprazole, metoclopramide, tamsulosin, terazosin, finasteride, meloxicam, lovastatin, and cyclobenzaprine.

### Based on bench data

Experimental studies investigating the role of medication ingredients in regulating bone hemostasis have been emerged. And the association found between OP diagnosis and compounds without prior clinical evidence may underlie their effects on bone remodeling, sympathetic regulation, and gastric acidity.

• Bone remodeling The balance between osteoclastogenesis and osteoblast expressing receptor activator of nuclear factor-kappa B ligand (RANKL) and osteoprotegerin (OPG) is regulated by systemic hormones, such as parathyroid hormone and local signaling molecules (Celebi Torabfam and Porsuk, 2024). Carisoprodol, a skeletal muscle relaxant, is associated with increased OP risk by inhibiting osteoblast differentiation and reducing bone density through inhibiting Wnt/beta-catenin signaling pathway (YRKM, 2022). Similarly, histamine promotes bone resorption by inducing osteoclast formation and increasing RANKL expression in osteoblasts and bone marrow cells (Ng et al., 2022; Biosse-Duplan et al., 2009). And histamine receptor H_1_ antagonists (brompheniramine and promethazine) and histamine receptor H_2_ antagonist (famotidine) contribute to protect against this (Abra et al., 2013). And the alpha-blocker, tamsulosin, exerts significant anti-osteoporotic effects by inhibiting the activity of transmembrane protein 16A (TMEM16A), which reduces the differentiation and function of osteoclasts, thereby decreasing bone resorption (Li et al., 2025).
• Sympathetic regulation The neurotransmitters norepinephrine (NE) and acetylcholine (Ach) released from the terminals of sympathetic and parasympathetic nerve fibers, respectively, in bone tissue can promote and inhibit neuropeptide Y (NPY) produced by osteocytes, thereby affecting the osteogenic differentiation of bone marrow stem cells (BMSCs) and OP development (Zhang et al., 2021). Thereby, bone loss in chronic heart failure underlies the adverse impact of the increased sympathetic tone on bone health (Guan et al., 2023). Herein, dicyclomine, oxybutynin, albuterol, and pseudoephedrine increase sympathetic nervous system (SNS) activity. Specifically, anticholinergics block Ach (Ogawa et al., 2021; Lerche, 2024), while albuterol and pseudoephedrine activate NE receptors, with albuterol binding to beta-adrenoceptors (Cardet et al., 2019) and pseudoephedrine stimulating alpha-1 adrenergic receptors (Perrin et al., 2015). In contract, the cardiovascular agents (losartan and quinapril) reduce SNS activity (Houglum et al., 2024).
• Gastric acidity A variety of enteroendocrine cells (EECs) distributed in the gastrointestinal tract, sensing external stimuli and regulating metabolism and behaviors by secreting various neuroendocrine peptides, and gut microbiota has been considered as a virtual endocrine organ (Huang et al., 2022). Thus, changes in the acidity of digestive system affects endocrine and body’s ability to absorb bone-boosting calcium (Chanpaisaeng et al., 2021). Therefore, the acid blockers, including H_2_ blocker (famotidine) and PPIs (pantoprazole and omeprazole), are commonly used in OP patients. Long-term use of PPIs has been reported to be associated with lower femoral neck BMD and a higher risk of OP (Fattahi et al., 2019).

Note that, OP increases infection risk and antibiotic use. For example, combination drug amoxicillin/clavulanate is often prescribed (Khan, 2023). Hence, a significant correlation of OP with only one specific anti-infective agent, clavulanate, was observed, which is due to the frequent prescription of amoxicillin in common conditions.

### Implications for practice and researchers

This study emphasizes the importance of a multidisciplinary approach for healthcare professionals in managing OP, particularly among the elderly with multiple health issues. Traditional anticonvulsants contribute to OP (LeBoff et al., 2022), while it has been reported in 2016 that the effect of new antiepileptic drugs such as gabapentin and topiramate on bone metabolism and bone density are scanty and controversial (Arora et al., 2016). The finding of this study revealed that pregabalin and gabapentin were still commonly prescribed in elder patients with OP diagnosis while topiramate was avoided. Moreover, the risk of OP varies with lovastatin dosage, i.e., lower doses (up to 10 mg daily) lows OP risk while higher doses increase the risk (Leutner et al., 2019), suggesting need the for examining cumulative drug exposure. This study underscores the urgent need for researchers to explore the mechanisms of action of medications in their intended conditions and OP development which lack of clinical and experimental supports and significantly associated with OP diagnosis, including terazosin, meloxicam, finasteride, metoclopramide, and cyclobenzaprine.

### Limitations

This study has several limitations. 1) Data of 2011–2012 and 2015–2016 OP questionnaires are not collected, limiting the continuity in national trend analysis from 1999 to March 2020. 2) The cross-sectional design limits the ability to establish causality of disease occurrence and medication intake with OP. It also restricts the evaluation of cumulative drug exposure on OP risk, given that OP is a chronic condition (Foessl et al., 2023). 3) There was a potential bias due to the exclusion of participants with missing data. 4) Though the survey staffs aimed to capture all prescription medication use, underreporting is possible, and data on most over-the-counter medications were not collected. 5) Lack of data on medication adherence, which may affect the interpretation of medication use. 6) Reliance on self-reported data for key variables introduces a risk in the recall and social desirability. 7) Limited fracture history data, focusing on self-reported hip, wrist, and vertebral fractures. 8) Residual confounding may arise from unmeasured variables, including smoking, physical activity, diet, and comorbid conditions.

## Conclusion

This study illustrates the association between OP clinical diagnosis and medication prescribed, pointing out aspects that need attention in clinical practice to prevent drug-induced OP in treat elderly with multi-comorbidities. Medication containing ingredients that pose risks for OP should be closely monitored in populations susceptible to the condition. Moreover, it has been found in this study that 15 medication ingredients significantly associated with OP diagnosis were lack of clinical support, amongst five with unclear mechanisms of actin in regulating bone homeostasis. And collaborative efforts between clinicians and researchers are vital for developing evidence-based guidelines to navigate the complexities of treating OP, especially in the context of polypharmacy.

## Data Availability

The original contributions presented in the study are included in the article/Supplementary Material, further inquiries can be directed to the corresponding authors.

## Abbreviations

ACEI, angiotensin-converting enzyme inhibitor; AR, 5-alpha reductase; BMD, bone mineral density; BMI, body mass index; CDC, Centers for Disease Control and Prevention; CI, confidence interval; DEXA, dual-energy X-ray absorptiometry; NHANES, National Health and Nutrition Examination Survey; NSAID, non-steroidal anti-inflammatory drug; OP, osteoporosis; OR, odds ratio; PIR, family income to poverty ratio; SNS, sympathetic nervous system; STROBE, Strengthening the Reporting of Observational Studies in Epidemiology.
