# Supplementary material for: Osteoporosis diagnosis and ingredients of prescription medications: a population-based study
Source: Front Pharmacol. 2025 Jul 11;16:1522937. doi: 10.3389/fphar.2025.1522937 (PMC12289469; doi:10.3389/fphar.2025.1522937)
Supplement: Supplementary file 1 [file Supplementaryfile1.docx]

**Supplemental tables**

**Table S1.** Clinical, demographic, and socioeconomic characteristics of participants in this study.^a^

| ***Unweight sample, No.*** | | 21965 |
| --- | --- | --- |
| ***Weighted sample, No.^b^*** | | 669561146 |
| ***Osteoporosis diagnosis*** | Yes | 11.06 (10.38–11.75) |
|  | No | 88.94 (88.25–89.62) |
| ***Bone fracture history*** | Yes | 14.69 (13.91–15.46) |
|  | No | 85.31 (84.54–86.09) |
| ***Age*** | 50–59 | 42.36 (41.20–43.52) |
|  | 60–69 | 29.53 (28.55–30.52) |
|  | 70–79 | 18.54 (17.88–19.19) |
|  | ≥ 80 | 9.57 (9.00–10.14) |
| ***Sex*** | Male | 46.61 (45.98–47.24) |
|  | Female | 53.39 (52.76–54.02) |
| ***Race/ethnicity*** | Non-Hispanic white | 76.31 (73.83–78.78) |
|  | Non-Hispanic black | 9.73 (8.39–11.07) |
|  | Hispanic | 8.45 (7.00–9.90) |
|  | Other | 5.51 (4.73–6.28) |
| ***BMI, kg/m^2^*** | Underweight < 18.5 | 3.36 (2.96–3.76) |
|  | Normal weight 18.5–24.9 | 24.79 (23.88–25.71) |
|  | Overweight 25–29.9 | 35.17 (34.25–36.08) |
|  | Obese ≥ 30 | 36.68 (35.49–37.86) |
| ***Self-reported health status*** | Excellent or very good | 43.64 (42.22–45.07) |
|  | Good | 33.63 (32.68–34.58) |
|  | Fair or poor | 22.72 (21.57–23.88) |
| ***No. prescription medications*** | 1–3 | 37.03 (35.72–38.34) |
|  | 4–7 | 37.97 (36.98–38.95) |
|  | ≥ 8 | 25.00 (23.85–26.15) |
| ***Days taking medications*** | 0/refused/missing | 23.02 (22.12-23.93) |
|  | < 500 | 23.86 (22.90-24.82) |
|  | ≥ 500 | 53.12 (51.87-54.36) |
| ***Education level*** | < High school | 19.78 (18.38–21.17) |
|  | High school | 26.03 (24.71–27.35) |
|  | > High school | 54.19 (52.37–56.02) |
| ***PIR^c^*** | < 1 | 17.56 (16.45–18.67) |
|  | 1–2 | 18.86 (17.73–20.00) |
|  | 2–3 | 14.62 (13.75–15.49) |
|  | 3–4 | 12.39 (11.54–13.23) |
|  | ≥ 4 | 36.57 (34.77–38.36) |
| ***Insurance status*** | Insured | 91.11 (90.30–91.93) |
|  | Uninsured | 8.89 (8.07–9.70) |

^a^Data from NHANES. Data are present as prevalence, % (95% CI) unless indicated otherwise. ^b^Data are weighted to be nationally representative. ^c^Total family income divided by the poverty threshold.

**Table S2.** Sociaeconomic characteristics by osteoporosis diagnosis among US adults aged 50 and older, 1999–March 2020^a^

| **Characteristic** | **% (95% CI)** | | **p-value^b^** |
| --- | --- | --- | --- |
|  | **OP** | **Non-OP** |  |
| ***Race/ethnicity*** | | | |
| Non-Hispanic white | 82.25 (79.55–84.95) | 75.57 (73.05–78.09) | <.0001 |
| Non-Hispanic black | 5.53 (4.45–6.61) | 10.26 (8.84–11.67) |  |
| Hispanic | 7.11 (5.45–8.77) | 8.62 (7.16–10.08) |  |
| Other | 5.11 (3.83–6.39) | 5.56 (4.78–6.33) |  |
| ***Education level*** | | | |
| < High school | 20.79 (18.08–23.49) | 19.65 (18.27–21.03) | 0.00 |
| High school | 29.24 (26.75–31.74) | 25.63 (24.25–27.02) |  |
| > High school | 49.97 (46.93–53.01) | 54.72 (52.87–56.57) |  |
| ***PIR^c^*** | | | |
| < 1 | 19.98 (17.91–22.05) | 17.26 (16.12–18.39) | <.0001 |
| 1–2 | 24.18 (21.73–26.63) | 18.20 (17.09–19.32) |  |
| 2–3 | 17.36 (15.30–19.42) | 14.28 (13.40–15.16) |  |
| 3–4 | 12.08 (10.15–14.01) | 12.43 (11.56–13.30) |  |
| ≥ 4 | 26.40 (23.62–29.17) | 37.83 (35.99–39.68) |  |
| ***Insurance status*** | | | |
| Insured | 96.18 (95.02–97.34) | 90.48 (89.62–91.35) | <.0001 |
| Uninsured | 3.82 (2.66–4.98) | 9.52 (8.65–10.38) |  |

^a^Data from NHANES. Data are present as prevalence, % (95% CI) unless indicated otherwise. ^b^Calculated with χ² test to determine the consistency of categorical distribution of variables between OP and non-OP groups. ^c^Total family income divided by the poverty threshold.

**Table S3.** Trends in osteoporosis diagnosis by bone fracture history, No. prescription medication use, and days taking prescription medications among US adults aged 50 and older, 1999–2020^a^

| **Characteristic** | **Prevalence, % (95% CI)** | | | | | **p-trend**^b^ |
| --- | --- | --- | --- | --- | --- | --- |
|  | **1999–2002** (n = 4426) | **2003–2006** (n = 4431) | **2007–2010** (n = 5844) | **2013–2014** (n = 2693) | **2017–2020** (n = 4571) |  |
| ***Osteoporosis diagnosis*** | 9.00 (7.83–10.17) | 11.78 (10.31–13.25) | 10.75 (9.44–12.06) | 10.85 (8.93–12.76) | 13.23 (11.57–14.89) | 0.00 |
| ***Bone fracture history*** | | | | | | |
| No | 97.31 (96.80–97.83) | 97.63 (97.16–98.10) | 97.15 (96.22–98.09) | 96.16 (95.32–97.00) | 97.64 (97.06–98.21) | 0.00 |
| Yes | 1.87 (1.43–2.32) | 2.69 (2.17–3.20) | 2.37 (1.90–2.84) | 2.85 (1.91–3.78) | 3.84 (3.00–4.68) | 0.00 |
| ***No. prescription medications*** | | | | | | |
| 1–3 | 2.36 (1.79–2.94) | 2.59 (1.95–3.24) | 2.34 (1.78–2.90) | 2.38 (1.56–3.20) | 2.59 (1.76–3.42) | 0.97 |
| 4–7 | 3.11 (2.42–3.79) | 4.43 (3.58–5.27) | 4.08 (3.40–4.76) | 3.40 (2.70–4.09) | 4.82 (3.79–5.84) | 0.02 |
| ≥ 8 | 2.48 (1.94–3.01) | 3.97 (3.18–4.75) | 3.53 (2.71–4.36) | 4.13 (2.66–5.61) | 5.02 (3.95–6.09) | 0.00 |
| ***Days taking medications*** | | | | | | |
| < 500 | 3.11 (2.47-3.75) | 3.80 (2.83-4.77) | 2.81 (2.06-3.56) | 2.64 (1.69-3.59) | 2.40 (1.76-3.04) | 0.16 |
| ≥ 500 | 4.72 (3.86-5.57) | 7.06 (6.16-7.97) | 7.09 (6.14-8.05) | 7.20 (5.46-8.95) | 9.86 (8.29-11.43) | <.0001 |

^a^Data from NHANES. Data are weighted to be nationally representative. ^b^The p-trend was obtained using linear regression, and the combined cycle is considered a continuous variable.

**Table S4.** Weighted trends in the administration of prescription medication ingredients related to osteoporosis diagnosis among US adults aged 50 and older, 1999–2020^a^

| **Prescription medication^b,c,d^** | **Prevalence, % (95% CI)** | | | | | **p-trend**^e^ |
| --- | --- | --- | --- | --- | --- | --- |
|  | **1999–2002** (n = 4426) | **2003–2006** (n = 4431) | **2007–2010** (n = 5844) | **2013–2014** (n = 2693) | **2017–2020** (n = 4571) |  |
| ***CENTRAL NERVOUS SYSTEM AGENTS*** | | | | | | |
| Carisoprodol | 0.12 (0.02–0.21) | 0.56 (0.23–0.88) | 0.39 (0.15–0.64) | 0.28 (0.05–0.51) | 0.07 (0.01–0.13) | 0.00 |
| Cyclobenzaprine | 0.66 (0.37–0.94) | 0.97 (0.62–1.32) | 1.18 (0.82–1.54) | 1.49 (0.91–2.07) | 1.29 (0.87–1.72) | 0.01 |
| Pregabalin | 0.00 (0.00-0.00) | 0.09 (0.02–0.16) | 0.78 (0.45–1.10) | 0.85 (0.31–1.39) | 0.65 (0.40–0.91) | <.0001 |
| Topiramate | 0.05 (-0.05–0.14) | 0.54 (0.23–0.85) | 0.38 (0.15–0.61) | 0.78 (0.28–1.27) | 0.59 (0.22–0.96) | <.0001 |
| Gabapentin | 1.08 (0.68–1.48) | 2.08 (1.46–2.69) | 2.52 (1.92–3.13) | 4.14 (2.65–5.63) | 5.76 (4.65–6.87) | <.0001 |
| Oxycodone | 0.49 (0.25–0.74) | 0.94 (0.58–1.31) | 1.29 (0.76–1.82) | 1.72 (0.74–2.69) | 1.72 (0.97–2.46) | 0.00 |
| ***GASTROINTESTINAL AGENTS*** | | | | | | |
| Famotidine | 0.47 (0.23–0.71) | 0.58 (0.29–0.87) | 1.02 (0.66–1.37) | 1.11 (0.58–1.63) | 1.20 (0.83–1.58) | 0.01 |
| Metoclopramide | 0.63 (0.30–0.97) | 0.36 (0.11–0.61) | 0.42 (0.22–0.63) | 0.47 (0.08–0.87) | 0.21 (0.09–0.33) | 0.10 |
| Pantoprazole | 0.59 (0.13–1.06) | 2.66 (1.90–3.42) | 1.69 (1.26–2.13) | 3.47 (2.56–4.37) | 3.58 (2.81–4.35) | <.0001 |
| Omeprazole | 4.79 (3.79-5.79) | 6.30 (5.20-7.40) | 10.66 (9.22-12.10) | 12.86 (10.88-14.85) | 10.62 (8.86-12.38) | <.0001 |
| Dicyclomine | 0.27 (0.09–0.45) | 0.41 (0.18–0.63) | 0.40 (0.11–0.69) | 0.27 (-0.01–0.55) | 0.57 (0.19–0.95) | 0.55 |
| Oxybutynin | 0.53 (0.26–0.81) | 0.96 (0.64–1.29) | 0.76 (0.50–1.01) | 1.07 (0.53–1.61) | 1.20 (0.85–1.54) | 0.05 |
| ***CARDIOVASCULAR AGENTS*** | | | | | | |
| Losartan | 2.29 (1.59–2.98) | 2.56 (1.87–3.25) | 3.33 (2.67–3.99) | 7.11 (5.60–8.63) | 10.58 (9.05–12.11) | <.0001 |
| Quinapril | 1.31 (0.81–1.82) | 1.69 (1.18–2.19) | 1.09 (0.71–1.47) | 0.96 (0.30–1.62) | 0.27 (0.04–0.51) | <.0001 |
| ***GENITOURINARY TRACT AGENTS*** | | | | | | |
| Tamsulosin | 0.76 (0.47–1.06) | 1.64 (1.20–2.08) | 1.87 (1.45–2.29) | 3.15 (2.25–4.05) | 3.67 (2.92–4.43) | <.0001 |
| Terazosin | 1.46 (1.16–1.76) | 1.17 (0.84–1.50) | 1.28 (0.86–1.71) | 1.07 (0.32–1.82) | 0.62 (0.28–0.96) | 0.00 |
| ***RESPIRATORY AGENTS*** | | | | | | |
| Pseudoephedrine | 1.26 (0.83–1.69) | 1.01 (0.64–1.37) | 0.61 (0.28–0.93) | 0.37 (-0.02–0.75) | 0.06 (-0.02–0.14) | <.0001 |
| Albuterol | 3.51 (2.78–4.23) | 3.77 (3.05–4.48) | 4.41 (3.56–5.25) | 4.61 (3.42–5.80) | 3.38 (2.47–4.30) | 0.08 |
| Promethazine | 0.18 (0.05–0.30) | 0.39 (0.16–0.62) | 0.36 (0.03–0.70) | 0.37 (-0.01–0.75) | 0.49 (-0.09–1.08) | 0.11 |
| Brompheniramine | 0.15 (-0.01–0.31) | 0.16 (-0.06–0.37) | 0.07 (-0.01–0.16) | 0.00 (0.00-0.00) | 0.01 (-0.01–0.03) | 0.05 |
| ***HORMONES/HORMONE MODIFIERS*** | | | | | | |
| Finasteride | 0.37 (0.18–0.55) | 0.60 (0.38–0.81) | 0.75 (0.48–1.02) | 1.29 (0.53–2.04) | 2.09 (1.34–2.84) | <.0001 |
| Furosemide | 4.84 (4.08–5.60) | 5.67 (4.90–6.43) | 5.51 (4.78–6.24) | 4.97 (3.69–6.24) | 4.73 (3.82–5.64) | 0.09 |
| Thyroid desiccated | 0.00 (0.00-0.00) | 0.40 (0.16–0.64) | 0.23 (0.04–0.42) | 0.33 (0.00–0.67) | 1.15 (0.46–1.85) | <.0001 |
| Levothyroxine | 9.07 (7.98–10.16) | 11.93 (10.46–13.40) | 11.52 (10.24–12.80) | 11.60 (9.85–13.34) | 12.33 (10.54–14.12) | 0.00 |
| Spironolactone | 0.81 (0.50–1.11) | 1.08 (0.61–1.55) | 1.07 (0.64–1.50) | 1.30 (0.76–1.85) | 1.51 (0.95–2.08) | 0.15 |
| Raloxifene | 1.34 (0.94–1.75) | 1.14 (0.80–1.47) | 1.07 (0.70–1.45) | 0.70 (0.39–1.00) | 0.15 (-0.01–0.32) | <.0001 |
| ***METABOLIC AGENTS*** | | | | | | |
| Lovastatin | 0.94 (0.54–1.34) | 2.17 (1.49–2.84) | 2.74 (2.11–3.37) | 2.33 (1.53–3.13) | 1.74 (1.05–2.44) | <.0001 |
| Alendronate | 2.02 (1.45–2.60) | 3.54 (2.94–4.13) | 2.65 (2.23–3.08) | 1.69 (0.98–2.40) | 1.52 (0.93–2.11) | <.0001 |
| Risedronate | 0.23 (0.06–0.40) | 1.43 (0.97–1.89) | 1.18 (0.83–1.52) | 0.19 (0.03–0.36) | 0.05 (0.00–0.10) | <.0001 |
| ***ANTINEOPLASTICS*** | | | | | | |
| Methotrexate | 0.37 (0.14–0.59) | 0.51 (0.29–0.74) | 0.85 (0.54–1.16) | 0.53 (0.12–0.95) | 0.58 (0.27–0.89) | 0.07 |
| ***TOPICAL AGENTS*** | | | | | | |
| Indomethacin | 0.41 (0.08–0.74) | 0.27 (0.05–0.50) | 0.22 (0.11–0.33) | 0.15 (-0.01–0.32) | 0.16 (0.04–0.28) | 0.60 |
| Meloxicam | 0.13 (-0.01–0.26) | 0.44 (0.15–0.73) | 1.08 (0.79–1.37) | 2.40 (1.43–3.36) | 3.04 (2.20–3.87) | <.0001 |
| Rofecoxib | 2.76 (2.03-3.48) | 1.56 (0.94-2.17) | 0.00 (0.00-0.00) | 0.00 (0.00-0.00) | 0.00 (0.00-0.00) | <.0001 |
| ***ANTI-INFECTIVES*** | | | | | | |
| Clavulanate | 0.24 (0.05–0.44) | 0.07 (0.01–0.14) | 0.09 (-0.01–0.19) | 0.15 (-0.01–0.30) | 0.27 (-0.08–0.63) | 0.38 |

^a^Data from NHANES. Data are weighted to be nationally representative. ^b^Name of the medication ingredients is based on the NHANES records. ^c^National trends of the corresponding ingredients in Table 2 are presented. ^d^The medication ingredients are categorized into therapeutic classes using the Multum Lexicon Plus drug database. ^e^The p-trend is obtained using a linear regression model adjusted for education level, family income to poverty ratio, and insurance status; the combined cycle is considered a continuous variable.

**Table S5.** Association between osteoporosis diagnosis and prescription medication ingredient administration in the US adults aged 50–79 years, 1999–2020^a,b,c^

| **Prescription medication**^d^ | | **OR (95% CI)** | **p-value** |
| --- | --- | --- | --- |
| ***CNS AGENTS*** | |  |  |
|  | ***Skeletal muscle relaxants*** |  |  |
|  | Carisoprodol | 3.00 (1.28–7.01) | 0.01 |
|  | Cyclobenzaprine | 2.45 (1.51–3.98) | 0.00 |
|  | ***Anticonvulsants***^e^ |  |  |
|  | Pregabalin | 1.97 (0.96–4.07) | 0.07 |
|  | Topiramate | 0.20 (0.10–0.40) | <.0001 |
|  | Gabapentin | 2.14 (1.52–3.02) | <.0001 |
|  | ***Narcotic analgesics*** |  |  |
|  | Oxycodone | 1.91 (1.15–3.17) | 0.01 |
| ***GASTROINTESTINAL AGENTS*** | |  |  |
|  | ***H2-blockers*** |  |  |
|  | Famotidine | 2.54 (1.47–4.39) | 0.00 |
|  | ***Prokinetics*** |  |  |
|  | Metoclopramide | 2.16 (1.08–4.33) | 0.03 |
|  | ***PPIs***^e^ |  |  |
|  | Pantoprazole | 1.46 (0.97–2.18) | 0.07 |
|  | Omeprazole | 1.22 (0.90–1.65) | 0.20 |
|  | ***Anticholinergics*** |  |  |
|  | Dicyclomine | 2.00 (0.97–4.14) | 0.06 |
|  | Oxybutynin | 2.03 (1.13–3.66) | 0.02 |
| **CARDIOVASCULAR AGENTS** | |  |  |
|  | ***ARBs*** |  |  |
|  | Losartan | 1.46 (1.09–1.96) | 0.01 |
|  | ***ACEIs*** |  |  |
|  | Quinapril | 0.46 (0.28–0.78) | 0.00 |
| ***GENITOURINARY TRACT AGENTS*** | |  |  |
|  | ***Alpha blockers*** |  |  |
|  | Tamsulosin | 0.44 (0.22–0.88) | 0.02 |
|  | Terazosin | 0.40 (0.20–0.81) | 0.01 |
| ***RESPIRATORY AGENTS*** | |  |  |
|  | ***Nasal decongestants*** |  |  |
|  | Pseudoephedrine | 0.29 (0.10–0.81) | 0.02 |
|  | ***Bronchodilators*** |  |  |
|  | Albuterol | 1.60 (1.18–2.18) | 0.00 |
|  | ***Antihistamines*** |  |  |
|  | Promethazine | 5.98 (1.35–26.46) | 0.02 |
|  | Brompheniramine | 8.31 (1.39–49.78) | 0.02 |
| ***HORMONES/HORMONE MODIFIERS*** | |  |  |
|  | ***AR inhibitors*** |  |  |
|  | Finasteride | 0.33(0.14–0.80) | 0.01 |
|  | Furosemide | 1.34 (0.97–1.85) | 0.07 |
|  | ***Thyroid hormones***^e^ |  |  |
|  | Thyroid desiccated | 2.12 (0.55–8.26) | 0.28 |
|  | Levothyroxine | 1.77 (1.45–2.16) | <.0001 |
|  | ***Mineralocorticoid receptor antagonists*** |  |  |
|  | Spironolactone | 1.87 (1.07–3.29) | 0.03 |
|  | ***SERMs*** |  |  |
|  | Raloxifene | 6.74 (3.94–11.53) | <.0001 |
| ***METABOLIC AGENTS*** | |  |  |
|  | ***Statins***^f^ |  |  |
|  | Lovastatin | 1.84 (1.15–2.93) | 0.01 |
|  | ***Bone resorption inhibitors*** |  |  |
|  | Alendronate | 42.39 (29.91–60.07) | <.0001 |
|  | Risedronate | 23.58 (13.15–42.31) | <.0001 |
| ***ANTINEOPLASTICS***^e^ | |  |  |
|  | ***Antimetabolites*** |  |  |
|  | Methotrexate^e^ | 2.19 (0.99–4.86) | 0.05 |
| ***TOPICAL AGENTS*** | |  |  |
|  | ***NSAIDs*** |  |  |
|  | Indomethacin | 0.15 (0.04–0.53) | 0.00 |
|  | Meloxicam | 1.91 (1.21–3.01) | 0.01 |
|  | Rofecoxib | 2.63 (1.52–4.53) | 0.00 |
| ***ANTI-INFECTIVES*** | |  |  |
|  | ***Beta-lactamase inhibitors*** |  |  |
|  | Clavulanate | 8.38 (1.65–42.44) | 0.01 |

^a^Data from NHANES. ^b^OR (95% CI) and p-value of the corresponding ingredients in Table 2 are present. ^c^Sensitivity analysis is performed by excluding elderly aged ≥ 80 years. ^d^The medication ingredients are categorized into therapeutic classes using the Multum Lexicon Plus drug database. ^e^Listed as a risk factor in the OP guideline. ^f^Statins are also known as HMG-CoA reductase inhibitors.

**Table S6.** Association between osteoporosis diagnosis and prescription medication ingredient administration in the non-obese US adults, 1999–2020^a,b,c^

| **Prescription medication**^d^ | | **OR (95% CI)** | **p-value** |
| --- | --- | --- | --- |
| ***CNS AGENTS*** | |  |  |
|  | ***Skeletal muscle relaxants*** |  |  |
|  | Carisoprodol | 1.99 (0.66–5.95) | 0.22 |
|  | Cyclobenzaprine | 2.08 (1.11–3.89) | 0.02 |
|  | ***Anticonvulsants***^e^ |  |  |
|  | Pregabalin | 2.25 (0.94–5.36) | 0.07 |
|  | Topiramate | 0.09 (0.02–0.37) | 0.00 |
|  | Gabapentin | 1.86 (1.21–2.87) | 0.01 |
|  | ***Narcotic analgesics*** |  |  |
|  | Oxycodone | 3.40 (2.00–5.77) | <.0001 |
| ***GASTROINTESTINAL AGENTS*** | |  |  |
|  | ***H2-blockers*** |  |  |
|  | Famotidine | 2.58 (1.44–4.63) | 0.00 |
|  | ***Prokinetics*** |  |  |
|  | Metoclopramide | 2.19 (0.95–5.06) | 0.07 |
|  | ***PPIs***^e^ |  |  |
|  | Pantoprazole | 1.58 (1.06–2.35) | 0.03 |
|  | Omeprazole | 1.41 (1.05–1.88) | 0.02 |
|  | ***Anticholinergics*** |  |  |
|  | Dicyclomine | 2.53 (1.14–5.62) | 0.02 |
|  | Oxybutynin | 3.64 (1.90–6.98) | 0.00 |
| **CARDIOVASCULAR AGENTS** | |  |  |
|  | ***ARBs*** |  |  |
|  | Losartan | 1.35 (0.97–1.88) | 0.08 |
|  | ***ACEIs*** |  |  |
|  | Quinapril | 0.57 (0.32–1.01) | 0.05 |
| ***GENITOURINARY TRACT AGENTS*** | |  |  |
|  | ***Alpha blockers*** |  |  |
|  | Tamsulosin | 0.45 (0.21–0.93) | 0.03 |
|  | Terazosin | 0.34 (0.15–0.79) | 0.01 |
| ***RESPIRATORY AGENTS*** | |  |  |
|  | ***Nasal decongestants*** |  |  |
|  | Pseudoephedrine | 0.37 (0.13–1.03) | 0.06 |
|  | ***Bronchodilators*** |  |  |
|  | Albuterol | 1.32 (0.84–2.06) | 0.22 |
|  | ***Antihistamines*** |  |  |
|  | Promethazine | 5.73 (0.95–34.72) | 0.06 |
|  | Brompheniramine | 17.70 (3.55–88.15) | 0.00 |
| ***HORMONES/HORMONE MODIFIERS*** | |  |  |
|  | ***AR inhibitors*** |  |  |
|  | Finasteride | 0.37 (0.14–0.95) | 0.04 |
|  | Furosemide | 1.19 (0.82–1.73) | 0.36 |
|  | ***Thyroid hormones***^e^ |  |  |
|  | Thyroid desiccated | 2.55 (0.67–9.68) | 0.17 |
|  | Levothyroxine | 2.03 (1.62–2.54) | <.0001 |
|  | ***Mineralocorticoid receptor antagonists*** |  |  |
|  | Spironolactone | 1.55 (0.83–2.90) | 0.17 |
|  | ***SERMs*** |  |  |
|  | Raloxifene | 8.20 (4.94–13.60) | <.0001 |
| ***METABOLIC AGENTS*** | |  |  |
|  | ***Statins***^f^ |  |  |
|  | Lovastatin | 1.51 (0.94–2.41) | 0.09 |
|  | ***Bone resorption inhibitors*** |  |  |
|  | Alendronate | 38.97 (26.72–56.82) | <.0001 |
|  | Risedronate | 20.03 (11.37–35.29) | <.0001 |
| ***ANTINEOPLASTICS***^e^ | |  |  |
|  | ***Antimetabolites*** |  |  |
|  | Methotrexate^e^ | 1.48(0.55–3.96) | 0.44 |
| ***TOPICAL AGENTS*** | |  |  |
|  | ***NSAIDs*** |  |  |
|  | Indomethacin | 0.14 (0.01–1.36) | 0.09 |
|  | Meloxicam | 1.52 (0.92–2.50) | 0.10 |
|  | Rofecoxib | 2.78 (1.66–4.66) | 0.00 |
| ***ANTI-INFECTIVES*** | |  |  |
|  | ***Beta-lactamase inhibitors*** |  |  |
|  | Clavulanate | 1.72 (0.27–10.89) | 0.56 |

^a^Data from NHANES. ^b^OR (95% CI) and p-value of the corresponding ingredients in Table 2 are present. ^c^ Sensitivity analysis is performed by excluding elderly with a BMI ≥ 30 kg/m^2^. ^d^The medication ingredients are categorized into therapeutic classes using the Multum Lexicon Plus drug database. ^e^Listed as a risk factor in the OP guideline. ^f^Stains are also known as HMG-CoA reductase inhibitors.
